# The peroxisome protein translocation machinery is developmentally regulated in the fungus *Podospora anserina*

**DOI:** 10.1128/spectrum.02139-23

**Published:** 2023-12-13

**Authors:** Beatriz Aguirre-López, Fernando Suaste-Olmos, Leonardo Peraza-Reyes

**Affiliations:** 1 Departamento de Bioquímica y Biología Estructural, Instituto de Fisiología Celular, Universidad Nacional Autónoma de México, Coyoacán, Mexico; Centro de Investigaciones Biologicas CSIC, Madrid, Spain

**Keywords:** peroxisomes, meiosis, sexual development, organelle protein import, filamentous fungi, *Podospora*

## Abstract

Peroxisomes are versatile organelles that are essential for diverse developmental processes. Peroxisome function critically depends on its matrix proteins, which are imported from the cytosol by the receptors Pex5 and Pex7. These receptors cycle into and out of peroxisomes through two membrane protein channels. Cargo-loaded Pex5 is imported into peroxisomes through the docking/translocation machinery, which is composed of Pex13 and Pex14. After cargo delivery, Pex5 is exported through the peroxisome ubiquitin ligase complex. This complex also ubiquitinates the receptors dictating their membrane extraction by the peroxisome AAA ATPase complex. In the fungus *Podospora anserina,* peroxisomes are required for meiotic induction. This process relies on PEX13 but not on PEX14. In addition, PEX14 is partially dispensable for peroxisome protein import during meiotic development, suggesting a developmental regulation of the translocation machinery. Here we show that PEX13 abundance in hyphae is maintained at low levels by the activity of the peroxisome ubiquitin ligase complex and the ubiquitin-conjugating enzyme PEX4, in conjunction with PEX14, PEX5, and with the peroxin that bridges the ubiquitination and the docking/translocation machineries, PEX8. Moreover, we found that the AAA ATPase complex and the peroxins that drive peroxisome membrane formation—PEX3 and PEX19—are also required to restrain PEX13 levels and that in their absence PEX13 associates with mitochondria. Furthermore, we found that PEX13 levels are higher throughout sexual development, where they are further increased during meiocyte and meiotic-spore differentiation. Our findings show that the peroxisome protein translocation machinery is subject to complex developmental regulation, which finely modulates PEX13 along sexual development.

**IMPORTANCE:**

Sexual reproduction allows eukaryotic organisms to produce genetically diverse progeny. This process relies on meiosis, a reductional division that enables ploidy maintenance and genetic recombination. Meiotic differentiation also involves the renewal of cell functioning to promote offspring rejuvenation. Research in the model fungus *Podospora anserina* has shown that this process involves a complex regulation of the function and dynamics of different organelles, including peroxisomes. These organelles are critical for meiosis induction and play further significant roles in meiotic development. Here we show that PEX13—a key constituent of the protein conduit through which the proteins defining peroxisome function reach into the organelle—is subject to a developmental regulation that almost certainly involves its selective ubiquitination-dependent removal and that modulates its abundance throughout meiotic development and at different sexual differentiation processes. Our results show that meiotic development involves a complex developmental regulation of the peroxisome protein translocation system.

## INTRODUCTION

Peroxisomes are versatile dynamic organelles that perform multiple cellular roles, including participation in essential metabolic pathways, such as fatty acid beta-oxidation and the glyoxylate cycle, as well as prominent roles in redox homeostasis and signaling (1 – 3). In addition, peroxisomes perform diverse metabolic functions, which vary among different organisms—glycolysis, the formation of biotin, siderophores, or of distinct secondary metabolites and signaling molecules—or even between different developmental stages or tissues (2, 4, 5). In addition, peroxisomes can act as scaffolds integrating complex signaling pathways, as for the immune response in animals (6). Moreover, peroxisome subcompartments can differentiate into separate organelles with particular functions, like the Woronin body that isolates hyphal compartments in ascomycete fungi (7).

Peroxisome function is importantly defined by the proteins residing in its matrix. The import of these proteins into the organelle is mediated by two conserved sorting pathways, which are driven by the cycling import receptors Pex5 and Pex7, respectively. These peroxins recognize the proteins to be imported into peroxisomes in the cytosol by binding to their peroxisomal targeting signals (PTS1 or PTS2, respectively) and are then docked at the peroxisome membrane by interactions with the docking/translocation complex. This complex is composed of the conserved peroxisome membrane proteins Pex13 and Pex14, and in fungi comprises additional components, such as Pex17 or Pex14/17 (Pex33) in yeast and mycelial fungi, respectively. Import of Pex7 (and its cognate cargo) in most eukaryotes requires binding to Pex5, whereas in fungi additional peroxins—referred to as PTS2 coreceptors (namely Pex20 or the yeast paralogous pair Pex18/Pex21)—participate in this process (8, 9). Cargo-loaded receptors are then translocated across the peroxisome membrane to be cycled back into the cytosol after releasing their cargo in the matrix (8, 10). It has been postulated that the translocation of PTS1 and PTS2 proteins across the membrane occurs through two independent channels, of which Pex14 is the main constituent (11, 12). On the other hand, recent evidence postulates that multiple Pex13 subunits, residing in the membrane with opposed orientations, conform a nuclear pore-like conduit, through which the cargo-loaded Pex5 crosses the membrane (13). Under this model, Pex14 could provide a binding site for Pex5 on the luminal side of peroxisomes through its N-terminal domain, to pull the cargo-loaded Pex5 out of the Pex13 conduit and propel its directional flow across the membrane (10, 13). Alternatively, it has been postulated that the protein import conduit consists of transient phase separation-driven Pex13/Pex14 condensates at the peroxisome membrane, to which cargo-loaded Pex5 partitions to release its cargo into the matrix (14). A second peroxisome membrane channel, composed of the RING-finger domain-containing peroxins Pex2, Pex10, and Pex12—the ubiquitin ligase complex (also referred to as the RING-finger complex)—drives the translocation of Pex5 back to the cytosol (15). The RING-finger complex also acts as ubiquitin (E3) ligase, which promotes the ubiquitination of the import receptors, a process required for peroxisome receptor export (15 – 17). Different types of ubiquitination dictate the fate of the receptors. While receptor recycling requires its monoubiquitination in a conserved cysteine near the protein N-terminus, the polyubiquitination of the receptors promotes their degradation *via* the proteasome. Both types of ubiquitination are facilitated by the peroxisome ubiquitin ligase complex but rely on different ubiquitin-conjugating (E2) enzymes. In yeasts, Pex4 (along with its membrane anchor/activator Pex22) catalyzes receptor monoubiquitination, while Ubc4 and Ubc5 conduct its polyubiquitination (16, 18). Ultimately, the ubiquitinated receptors are extracted from the membrane by the complex formed by the AAA ATPases Pex1 and Pex6 and their membrane anchor (Pex15 in yeasts or Pex26 in filamentous fungi and animals) (19 – 21).

In keeping with their functional versatility, peroxisomes are required for multiple developmental processes. Among them, many key aspects of the developmental processes that conduct sexual reproduction rely on peroxisomes (22 – 28). One such process is meiosis, the reductional division that allows for the alternation of the haploid/diploid phases of the sexual cycle and that promotes genetic recombination. We have shown that the initiation of meiotic development in the model ascomycete fungus *Podospora anserina* requires both peroxins implicated in peroxisome membrane formation, PEX3 and PEX19 (29), as well as most peroxins required for peroxisome matrix protein import (29 – 32). The exceptions are the import receptors PEX5 and PEX7 (33), as well as the docking peroxins PEX14 and PEX14/17 (29). Moreover, we discovered that PEX14 and PEX14/17 are not absolutely required for peroxisome matrix protein import at specific meiotic stages, namely during the differentiation of meiocytes (asci, a process occurring alongside meiotic prophase I in this fungus) and of the cellular products of meiosis (ascospores, the equivalents of gametes in animals), revealing that the functional state of the docking/translocation complex changes along meiotic development (29). Significantly, we demonstrated that the PTS2 coreceptor PEX20 and the docking peroxin PEX13 were absolutely required for meiosis initiation, in contrast to PEX5, PEX7, and PEX14. Moreover, we also demonstrated that PEX20’s function in this process depends on the conserved cysteine that is predictably monoubiquitinated to promote PEX20 recycling (29, 32). These observations lead us to propose the existence of a PEX20-driven import pathway required for meiotic induction in *P. anserina* and a foremost role for PEX13 in peroxisome protein import. Here, to better understand the regulation of the docking/translocation complex during meiotic development, we studied PEX13 localization and dynamics during *P. anserina* sexual cycle. Our findings show that PEX13 activity is subject to a complex developmental regulation, which implicates a differential modulation of its abundance along sexual development.

## RESULTS

### PEX13 levels in hyphae increase when the peroxisome ubiquitination complex is defective

**Fig 1:**
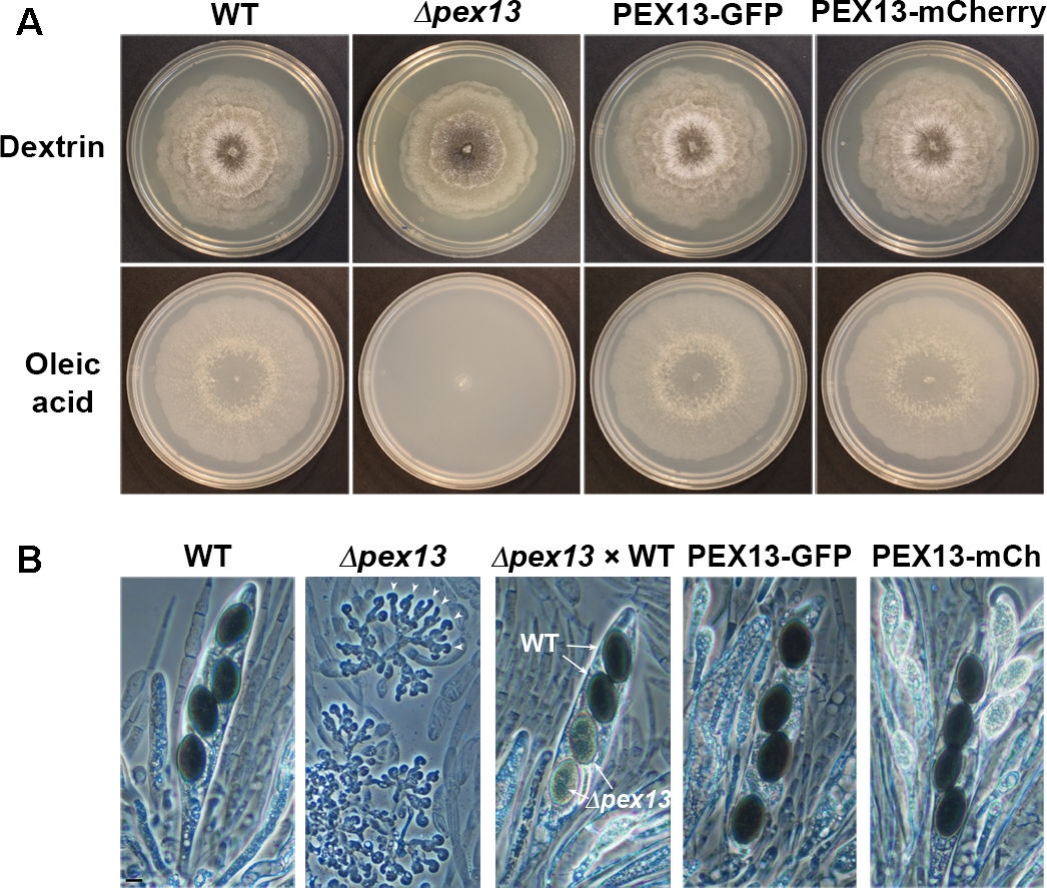
Phenotypes of *P. anserina* strains expressing fluorescent-labeled PEX13. (**A**) Mycelial colonies of wild-type (WT) and *PEX13*-deleted (*Δpex13*) strains, and of strains expressing GFP- or mCherry-labeled versions of PEX13 grown for 6 days in media containing dextrin (top) or oleic acid (bottom) as sole carbon source. *PEX13* deletion in *P. anserina* affects mycelial growth in a dextrin-based medium (top) and prevents it in an oleic acid-based medium (bottom). By contrast, PEX13-tagged strains displayed a wild-type phenotype. (**B**) Sexual cycle cells issued from homozygous crosses of wild-type or *Δpex13* strains, from a heterozygous cross of *Δpex13* to the wild type (*Δpex13* × WT) or from homozygous crosses of strains expressing PEX13-GFP or PEX13-mCherry. *P. anserina* produces asci containing four black (melanized) ascospores in wild-type sexual crosses. By contrast, no ascospores are formed in *Δpex13* homozygous crosses, which produce only hook-shaped dikaryotic cells (croziers, arrowheads) and are therefore sterile (29). This phenotype is recessive, as a *Δpex13* to WT heterozygous cross produces ascospores. However, the pigmentation of the *Δpex13* ascospores produced in this cross is defective (*Δpex13* × WT, lower spores). By contrast, homozygous crosses of PEX13-GFP- or PEX13-mCherry-expressing strains are fertile and produce normal black ascospores. Scale bar, 10 µm.

**Fig 2:**
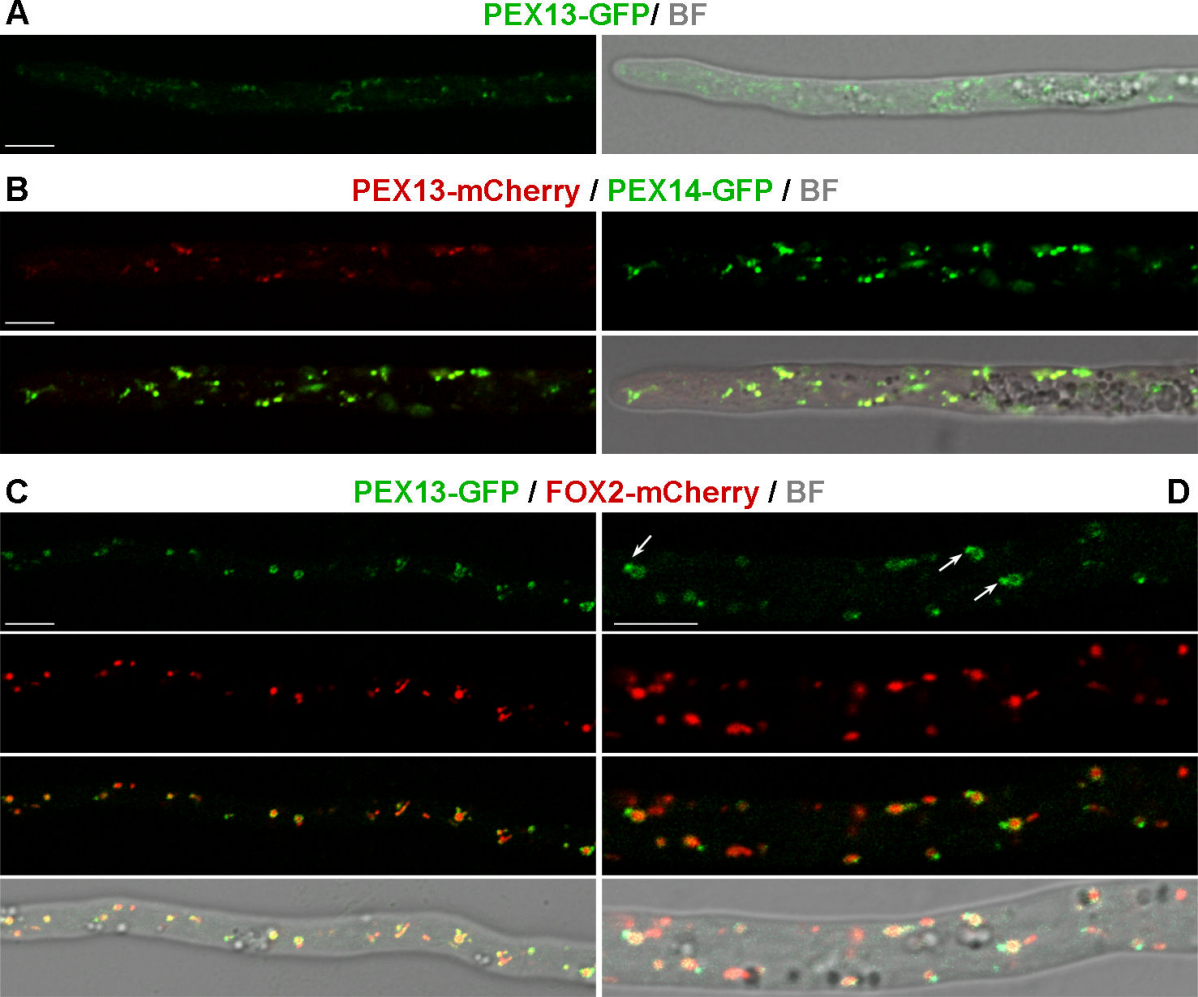
PEX13 localizes to peroxisomes in *P. anserina* hyphae. Confocal microscopy analysis of PEX13-GFP in hyphae (**A**) and compared localizations of PEX13-mCherry with PEX14-GFP (**B**), and of PEX13-GFP and FOX2-mCherry (**C-D**). Arrows point to small peroxisome buds enriched for PEX13. BF: bright field. Scale bar, 5 μm.

To study PEX13 localization, we generated strains expressing GFP or mCherry C-terminally tagged versions of PEX13, by tagging the *PEX13* gene at its native locus. Phenotypic analyses of these strains showed that PEX13 tagging did not affect its function (Fig. 1). We analyzed PEX13 localization in hyphae growing in standard dextrin-based media and observed that both PEX13 fluorescent protein fusions exhibited low fluorescence levels (Fig. 2). Nonetheless, PEX13 displayed punctate labeling, which in double-label experiments co-occurred with that of a fluorescent version of PEX14 (Fig. 2B) (Pearson’s correlation coefficient, *PCC*, avg. 0.79 ± 0.02, *n* = 21 hyphae), as well as with that of the peroxisome fatty acid β-oxidation multifunctional protein 2 (FOX2, a peroxisome matrix protein) (Fig. 2C) (*PCC*, avg. 0.63 ± 0.03, *n* = 21 hyphae), corroborating that PEX13 localizes to peroxisomes. We also observed that PEX13-GFP fluorescence was higher at the periphery of some peroxisomes (Fig. 2D), consistent with peroxisome membrane localization, and that this fluorescence often accumulated to small buds (arrows) of budding peroxisomes [i.e., in 96 of 141 (68%) observed budding peroxisomes, from 30 analyzed hyphae], suggesting that PEX13 accumulates in newly formed peroxisomes during peroxisome division.

**Fig 3:**
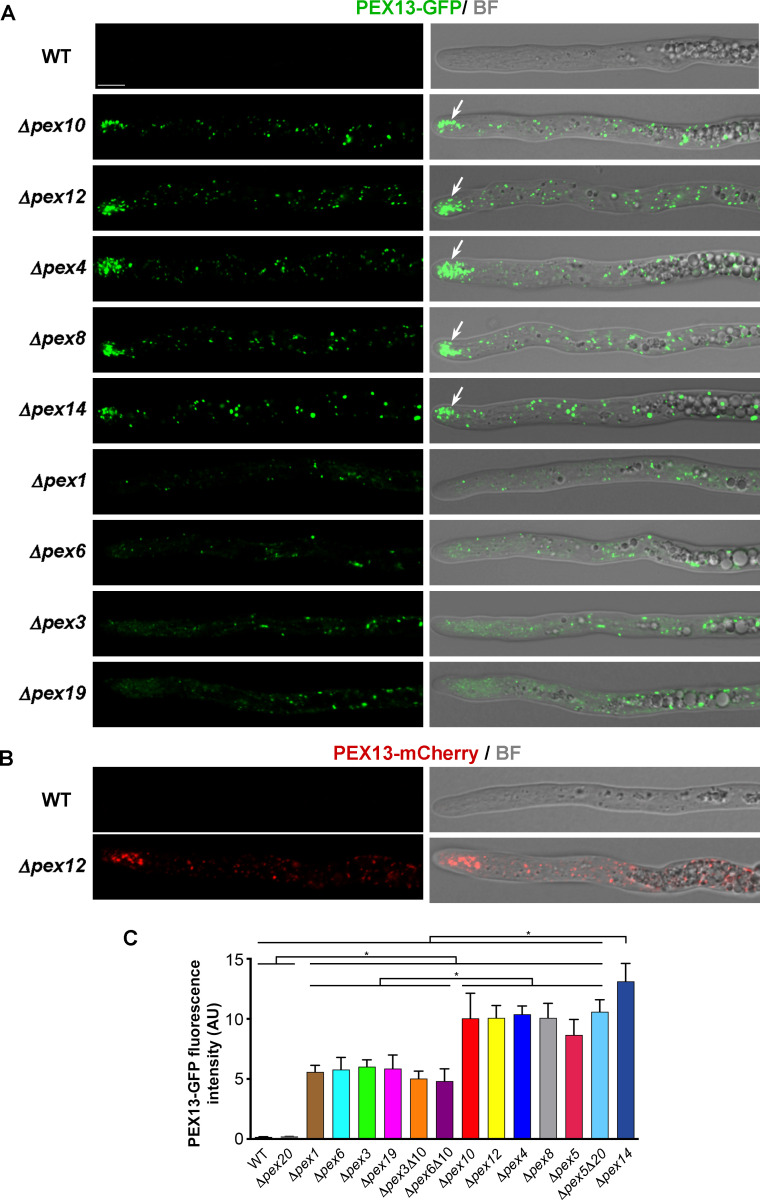
PEX13 localization in hyphae deficient for peroxisome biogenesis. Localization of PEX13-GFP (**A**) or PEX13-mCherry (**B**) in growing leading hyphae of the wild type (WT) and the indicated deletion mutant strains. Arrows point to peroxisome remnants accumulating at the hyphal tip. BF: bright field. Scale bar, 5 μm. Note that the imaging parameters differ from those of Fig. 2, where the detection was enhanced to visualize PEX13 fluorescence in the wild type. (**C**) Quantification of PEX13-GFP average fluorescence intensity in the apical region (i.e., in the region extending 80 μm from the tip) of growing leading hypha of the indicated strains. Histograms show the mean ± SD; *n* = 24 hyphae (from three independent experiments). * *P* < 0.05 for any pair of strains between the two overlined connected groups, by two-way ANOVA with Tukey’s post hoc test. AU, Arbitrary units.

In the ascomycete yeast *Hansenula polymorpha*, the steady-state levels of Pex13 increase when the RING-finger complex is defective, and it has been postulated that this complex facilitates the constitutive ubiquitination of Pex13 promoting its rapid degradation *via* the proteasome (34). We tested whether PEX13 was subject to similar regulation in *P. anserina* and analyzed PEX13 localization and abundance in strains deleted for *PEX10* or *PEX12*. We found that PEX13-GFP also displayed a punctate pattern in *Δpex10* or *Δpex12* hyphae (Fig. 3A). However, PEX13-GFP fluorescence intensity levels were dramatically increased in these strains, as compared to the wild type (Fig. 3A, quantitation in C). We also found that the disruption of the RING-finger complex produced a similar increase in the fluorescent levels of PEX13 when tagged with mCherry (tested for *Δpex12*, Fig. 3B), showing that these changes are not specific to the GFP-tagged protein. Next, we studied whether PEX4—the ortholog of the E2 enzyme catalyzing receptor monoubiquitination in yeasts (35 – 38)—was also involved in regulating PEX13 levels. We found that PEX13-GFP displayed a similar punctate pattern with similar fluorescence intensity in *Δpex4*, *Δpex10,* and *Δpex12* cells. In *P. anserina,* PEX10, PEX12, and PEX4 are required for peroxisome matrix protein import but not for peroxisome membrane formation (31, 32); thus, the PEX13-GFP punctate pattern of *Δpex10, Δpex12,* and *Δpex4* hyphae is consistent with a peroxisome-remnant localization for this protein in these cells. Of note, we also observed that several PEX13-labeled peroxisome remnants accumulated at the tip of *Δpex10, Δpex12,* and *Δpex4* hyphae (Fig. 3, arrows), which could indicate that the peroxisome protein-import and transport systems are related.

**Fig 4:**
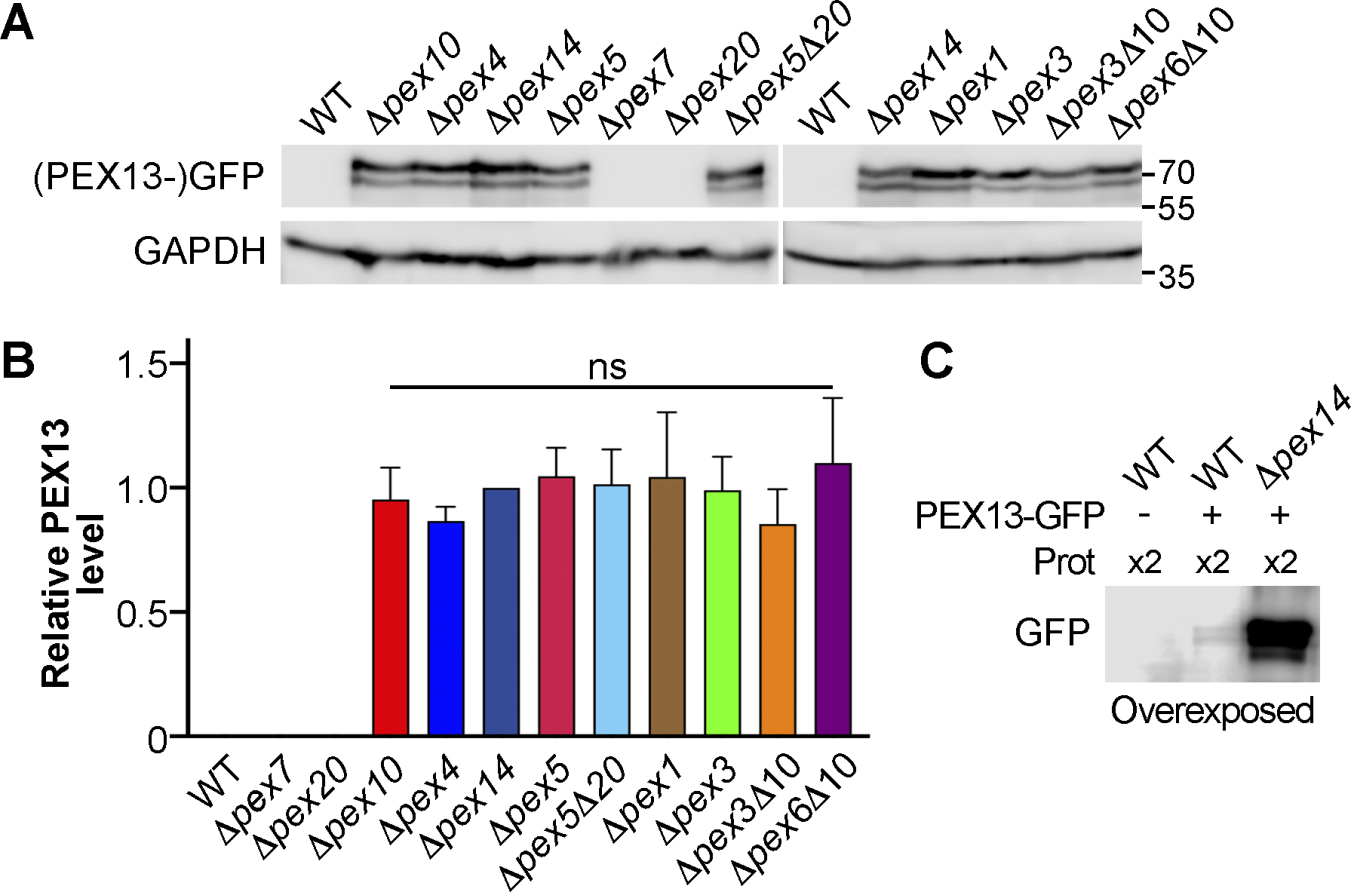
Effect of deletion of distinct peroxin genes on PEX13 mycelial steady-state levels. (**A**) Western blot analysis of total mycelial protein extracts of the indicated strains expressing PEX13-GFP using anti-GFP (top) or anti-GAPDH (bottom) antibodies. (**B**) Quantitation of PEX13 levels normalized to GAPDH, the value of each strain is relative to that of *Δpex14* samples, which were set to 1 (note that no signal is detected with these parameters in the wild type); ns, no statistically significant differences between the overlined groups were observed by one-way ANOVA with Tukey’s post hoc test, *n* = 3. (**C**) Western blot showing the detection of PEX13-GFP in the wild-type context. The first lane shows a negative control, corresponding to a wild-type strain where *PEX13* was not tagged with GFP. Note that increased amounts of total protein per sample were loaded and that the membrane was overexposed.

To corroborate that the deficiency of the peroxisome ubiquitination complex leads to an increase in PEX13 protein levels, we analyzed PEX13-GFP steady-state levels by Western blotting of mycelial protein extracts using an anti-GFP antibody. We found that the elimination of either PEX10 or PEX4 resulted in a significant increase in PEX13-GFP steady-state levels (Fig. 4A and B). Actually, PEX13-GFP could only be detected in wild-type samples by overexposure of the immunoblots (Fig. 4C). Of note, in addition to a major band detected with the anti-GFP antibody, whose mobility was consistent with the predicted molecular weight of PEX13-GFP (73.3 kDa), a second minor band of higher mobility was reproducibly detected. This protein band was always detected in correlation with the presence of PEX13-GFP (Fig. 4A and C) and might represent a cleaved product of this protein. These observations show that levels of PEX13 in *P. anserina* vegetative cells increase when the peroxisome ubiquitination complex is defective.

### PEX13 hyphal levels also increase when the docking complex is defective

Next, we studied the involvement in this process of the docking peroxin PEX14 and of PEX8—the peroxin that predictably links the RING-finger complex to the docking complex (39). We found that PEX13-GFP localization and fluorescence intensity in *Δpex8* and the ubiquitination-complex mutants were alike (Fig. 3A and C). In *Δpex14* hyphae, PEX13-GFP also displayed a punctate pattern and high fluorescence intensity. However, in this latter mutant, PEX13-GFP-labeled peroxisome remnants appeared to be larger and their hyphal tip accumulation was less profuse (Fig. 3A). Quantification of PEX13-GFP fluorescence intensity showed that it was significantly higher for *Δpex14* than for the other mutants (Fig. 3C). In addition, we found that *PEX14* deletion also resulted in a significant increase in PEX13-GFP steady-state protein levels (Fig. 4). By Western blotting, no significant differences between the protein levels of PEX13-GFP in *Δpex14* and the ubiquitination-complex mutants were detected (Fig. 4B).

### PEX13 associates with mitochondria in the absence of PEX3, PEX19, or the peroxisome AAA ATPase complex

**Fig 5:**
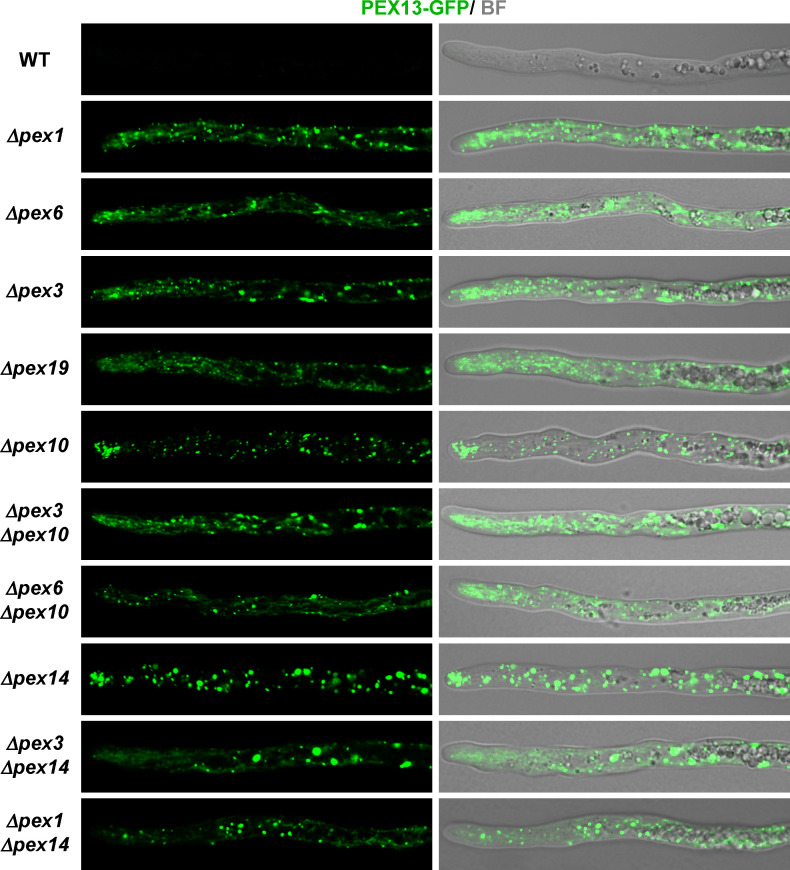
PEX13 localizes to a network of elongated cytoplasmic compartments in hyphae deficient for peroxisome membranes. PEX13-GFP localization in growing leading hyphae of the wild type and the indicated deletion mutant strains. BF: bright field. Scale bar, 5 μm. Note that the imaging parameters differ from those of Fig. 3, as detection was enhanced to visualize PEX13-GFP in the cytoplasmic compartments.

**Fig 6:**
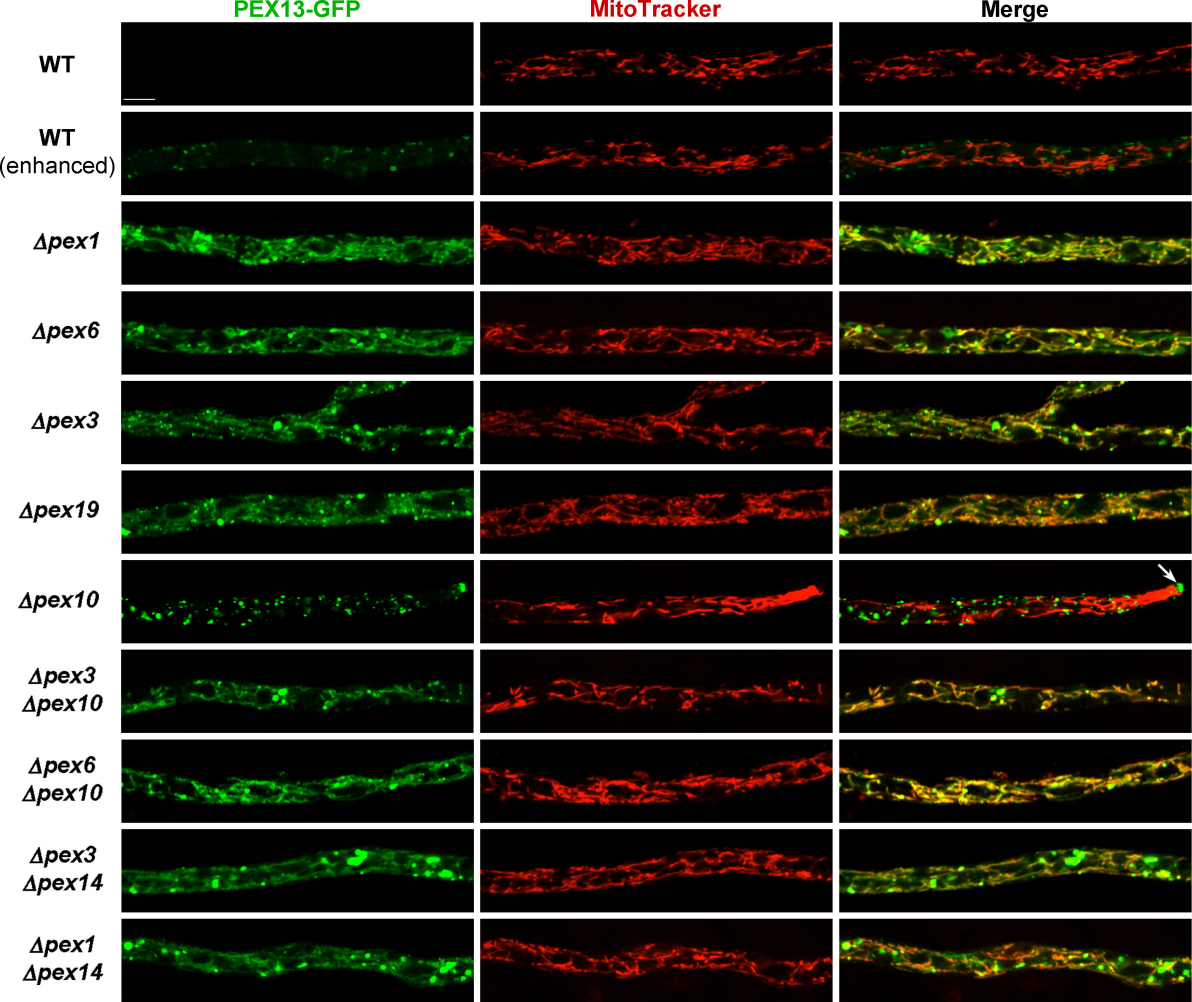
PEX13 associates with mitochondria in hyphae deficient for peroxisome membranes. Localization of PEX13-GFP in hyphae of the indicated genotypes stained by MitoTracker red. Excepting *Δpex10*, which shows the hyphal apex (arrow, apical peroxisome remnants), images show the subapical region (beyond 80 μm from the tip) of growing leading hyphae, BF: bright field. Scale bar, 5 μm. To allow PEX13 visualization in the wild type, the power of the GFP excitation laser was enhanced in the (*enhanced*) images.

We have previously shown that peroxisomes containing PEX14 are lost upon disruption of the peroxisome AAA ATPase complex (PEX1, PEX6, or PEX26), in a process involving their vacuolar removal likely by pexophagy (32). Actually, it is known that pexophagy is enhanced when the AAA ATPase complex is defective (40, 41). We analyzed whether PEX13 was subject to a similar regulation than PEX14 and studied its localization in the absence of PEX1 or PEX6. We found that PEX13-GFP exhibited fluorescent intensity levels in *Δpex1* and *Δpex6* hyphae that were significantly higher than in the wild type. However, it did not reach the levels observed in the RING finger or docking-complex mutants (Fig. 3A and C). We observed that PEX13-GFP exhibited a punctate pattern in *Δpex1* and *Δpex6* hyphae; however, we also observed a dimmer cytoplasmic PEX13 fluorescence in these cells (Fig. 3A). Further analyses revealed that PEX13-GFP punctae were embedded in, or in close proximity to, a network of PEX13-GFP-labeled elongated cytoplasmic structures (shown for apical and subapical cells in Fig. 5 and 6, respectively). Double-label experiments staining cells with MitoTracker Red indicated that these structures corresponded to mitochondria and showed that PEX13-GFP associates with these organelles when the peroxisome dislocation complex is defective (Fig. 6).

The association of PEX13 to mitochondria in *Δpex1* and *Δpex6* cells could be produced by the loss of peroxisome remnants/membranes that takes place when the AAA ATPase complex is defective (32). Therefore, we asked whether PEX13 was also associated with mitochondria in hyphae lacking PEX3 or PEX19, which are required for peroxisome membrane formation (42). We analyzed PEX13-GFP localization in *Δpex3* and *Δpex19* hyphae and found that it exhibited a very similar localization pattern and intensity levels to those of *Δpex1* and *Δpex6* hyphae (Fig. 3A, C, 5, and 6). Moreover, PEX13-GFP also colocalized with MitoTracker-stained mitochondria in *Δpex3* and *Δpex19* hyphae (Fig. 6).

In addition, we corroborated that disrupting the AAA ATPase complex (tested for *Δpex1*) or peroxisome membrane biogenesis (tested for *Δpex3*) also resulted in increased steady-state protein levels of PEX13 by Western blotting (Fig. 4A and B). Again, by this analysis, we did not detect differences in the steady-state levels of PEX13-GFP between these mutants and the ubiquitination- or docking-complex mutants. This might reflect the physiological heterogeneity along the mycelial colonies and could indicate that the differences in PEX13-GFP levels between these strains observed in leading hyphae by fluorescence imaging might only occur in the extension zone of the colonies.

Altogether, the above findings indicate that PEX13 localizes to mitochondria when peroxisome membranes are missing. In addition, these observations indicate that the presence of peroxisome membranes is required to maintain low levels of PEX13 in vegetative hyphae. Consistent with these interpretations, we found that either *Δpex6* and *Δpex3* are epistatic to *Δpex10* for the PEX13-GFP cell localization phenotype (Fig. 3C, 5, and 6) and that increased protein levels of PEX13 also accumulated in *Δpex3Δpex10* and *Δpex6Δpex10* mycelia (Fig. 4A and B). Moreover, we found that PEX13-GFP also localized to mitochondria in the double *Δpex1Δpex14* and *Δpex3Δpex14* mutants (Fig. 5 and 6). However, some punctae labeled by PEX13-GFP in these cells were larger than in the single *Δpex1* or *Δpex6* mutants.

### PEX13 hyphal levels increase upon loss of PEX5

**Fig 7:**
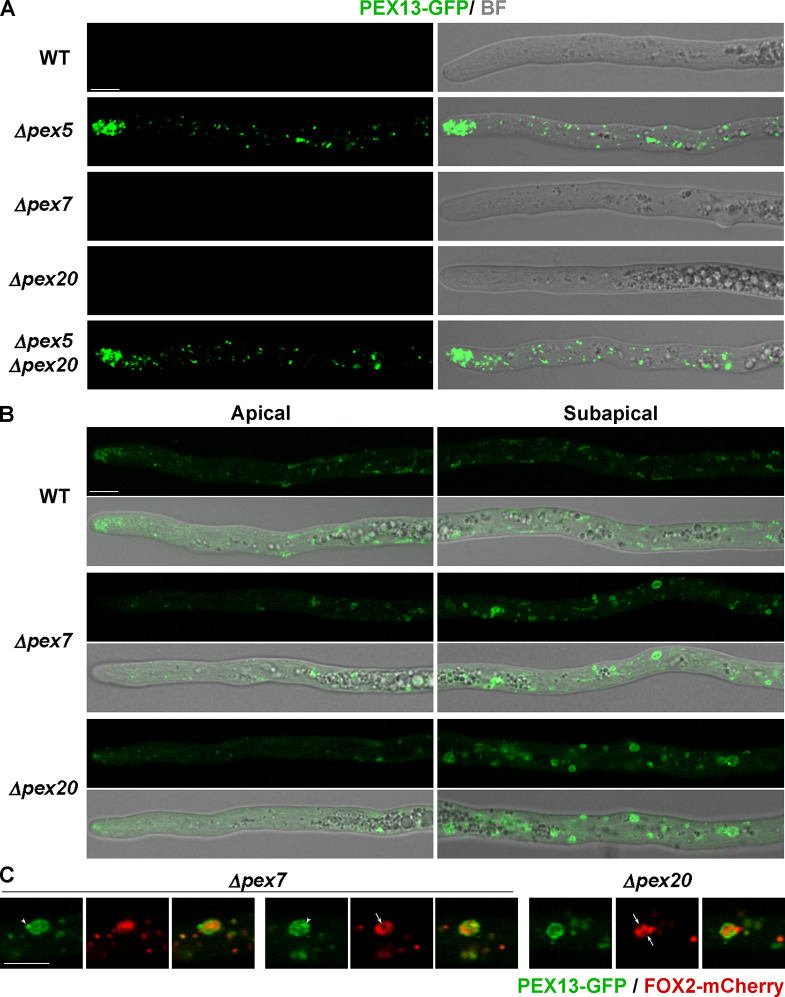
PEX13 localization in hyphae deficient for the peroxisome matrix protein import receptors. (**A**) Confocal microscopy analysis of PEX13-GFP in growing leading hyphae of strains of the indicated genotypes. (**B**) Localization of PEX13-GFP in the apical (left panels) and subapical (beyond 80 μm from the tip, right panels) hyphal regions of the indicated strains. Note that the imaging parameters in A and B differ. (**A**) is equivalent to Fig. 3A, whereas (**B**) is equivalent to Fig. 2A. (**C**) Compared localization of PEX13-GFP and FOX2-mCherry in subapical *Δpex7* and *Δpex20* cells. Note the foci enriched for PEX13-GFP (arrowheads) and the internal punctae devoid of FOX2-mCherry fluorescence (arrows). Scale bar, 5 μm.

PEX5, PEX7, and PEX20 are differentially involved in *P. anserina* development; therefore, we asked whether their elimination differentially affected PEX13 levels. We analyzed PEX13-GFP localization in *Δpex5, Δpex7,* and *Δpex20* hyphae and found that PEX5 elimination produced an increase in PEX13-GFP levels, which was comparable to that of the ubiquitination-complex mutants (Fig. 7A, compare to 3A, quantification in Fig. 3C). Likewise, PEX13-GFP displayed a punctate pattern in *Δpex5* hyphae with a cluster of peroxisomes accumulating at the tip, similar to that of the ubiquitination-complex mutants (Fig. 7A). In the apical segment of *Δpex7* and *Δpex20* leading hyphae (i.e., the first ≈ 80 μm behind the tip), PEX13-GFP labeling and levels were similar to the wild-type ones (Fig. 7A and B, quantified for *Δpex20* in Fig. 3C); however, PEX13-GFP decorated larger and brighter spherical cytoplasmic structures in the subapical segments of *Δpex7* and *Δpex20* hyphae, which were not observed in the wild type (Fig. 7B). Upon double labeling, the periphery of these structures was enriched for PEX13-GFP, which circumscribed an internal FOX2-mCherry labeling (Fig. 7C) (note that FOX2 import into peroxisomes in *P. anserina* depends on PEX5 and not on the PTS2 (co)receptors (29, 32)), indicating that they likely represent large “swollen” peroxisomes. However, FOX2-mCherry fluorescence was often excluded from small internal punctate areas (Fig. 7C, arrows), suggesting a complex internal organization. Consistent with the imaging analysis, we observed increased PEX13-GFP protein levels in *Δpex5* mycelia by Western blotting, but not in *Δpex7* or *Δpex20* (Fig. 4A and B); and we found that *Δpex5* was epistatic on *Δpex20* for these phenotypes (Fig. 3C, 4A and B, 7A). These findings show that the import receptor PEX5 is also involved in restraining PEX13 levels in vegetative cells.

### PEX13 is differentially regulated during sexual development

**Fig 8:**
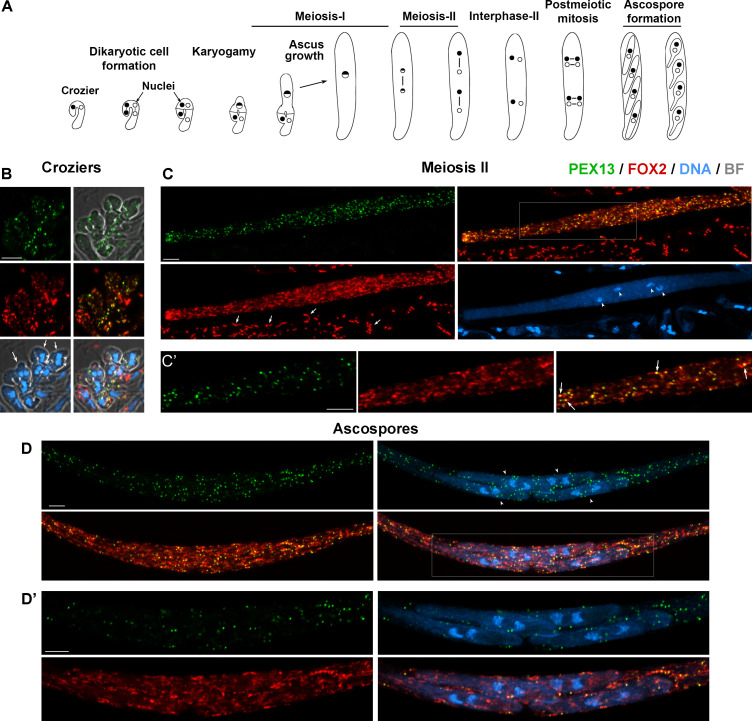
Localization of PEX13 and FOX2 during sexual development. (**A**) *P. anserina* sexual development from dikaryotic stage to ascospore formation (from left to right, see text for details). Compared localization of PEX13 and FOX2 in dikaryotic crozier cells (**B**), grown meiotic asci (C, here an ascus ending meiosis II) and early ascospores (**D**). (**C′**) and (**D**′) show single-plane magnifications of the boxed areas in (**C**) and (**D**), respectively. Images in B, C, and D show maximum-intensity projections of z-series through the entire cells. Arrows in (**B**) point to croziers, in (**C**) to FOX2-mCherry-labeled paraphysal peroxisomes (note the lack of detectable PEX13-GFP labeling) and in (**C′**) to PEX13-GFP foci decorating FOX2-labeled elongated peroxisomes. Arrowheads in (**B**) and (**C**) point to nuclei, and in (**D**) to ascospores (note the presence of two nuclei per ascospore). DNA was stained by Hoechst. Scale bar, 5 μm.

Next, we studied PEX13 abundance and distribution during sexual development. *P. anserina* sexual development (Fig. 8A) takes place inside multicellular fructifications, which result from the fertilization of the female gametangium (the ascogonium) and that are called perithecia. The ascogonial cells present inside these structures differentiate a specialized cell type called the crozier, where nuclei of opposite parental origin (and opposed mating type) are associated by pairs. The differentiation of this specialized hypha involves the asymmetrical extension of its apical region, producing a hook-shaped hypha. A pair of nuclei of different mating types is placed at the crook segment of the crozier, and after coordinated mitoses and septa formation, the four resulting nuclei are isolated in three cells: an upper binucleate dikaryotic cell and two flanking uninucleate cells. The dikaryotic cell undergoes karyogamy and differentiates into an ascus (the meiocyte) by extending from the upper crook region, while the uninucleate cells fuse and ultimately produce a new crozier. Karyogamy is immediately followed by meiosis, and the ascus then extends from ≈10 to over 150 μm along prophase I. Following ascus growth, the two meiotic divisions take place, and the four resulting nuclei undergo mitosis. New plasma membranes are then produced inside the ascus, which surrounds each pair of non-sister nuclei, yielding four new binucleate cells. These cells differentiate into ascospores while growing asymmetrically and increasing their volume about 10 times (43 – 45).

To study PEX13-GFP along sexual development, first we performed homozygous crosses between strains simultaneously expressing fluorescently labeled versions of PEX13 and FOX2. Analyses of sexual cycle cells issued from these crosses showed that, overall, PEX13-GFP colocalizes with FOX2-mCherry at all sexual development stages (shown for croziers, meiotic asci, and ascospores in Fig. 8B, C, and D, respectively), corroborating the peroxisome localization of PEX13 also during the sexual cycle. By studying the localization of peroxisome matrix-located proteins, we have previously shown that the morphology of peroxisomes changes along meiotic development, from spherical to elongated by the end of the first meiotic division and display this morphology until the early stages of ascospore formation (46 – 48). Interestingly, in contrast to this morphology, we observed that PEX13 displayed a punctate pattern at these developmental stages (shown for an ascus after meiosis II and for early ascospores in Fig. 8C and D, respectively). Nevertheless, PEX13-labeled punctae colocalized with FOX2 as discrete foci localizing along FOX2-labeled peroxisomes (Fig. 8C′ and D′, arrows). This indicates that PEX13 localizes to specific domains of the peroxisome membrane during late meiotic development.

**Fig 9:**
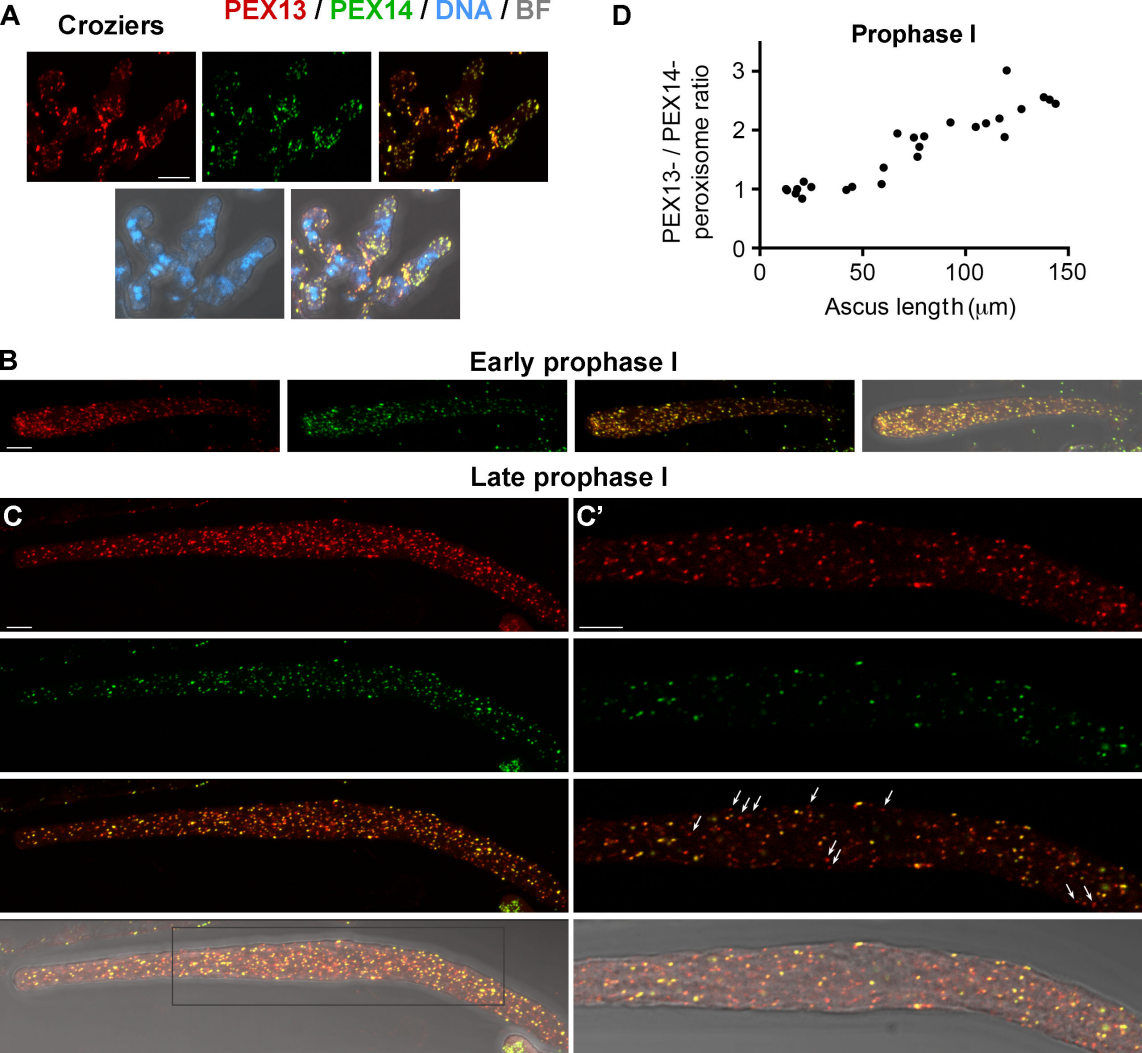
Localization of PEX13 and PEX14 during meiotic development. Compared localization of PEX13 and PEX14 in dikaryotic crozier cells (**A**), and in early (**B**) and late (**C**) meiotic prophase I asci. (**C′**) shows a single-plane magnification of the boxed area in (**C**). Images in A, B, and C show maximum-intensity projections of z-series through the entire cells. Arrows show examples of PEX13-mCherry-labeled peroxisomes with no detectable or highly reduced levels of PEX14-GFP. DNA was stained by Hoechst. Scale bar, 5 μm. (**D**) The ratio of PEX13-mCherry- to PEX14-GFP-labeled peroxisomes in prophase I asci in relation to the ascus length.

**Fig 10:**
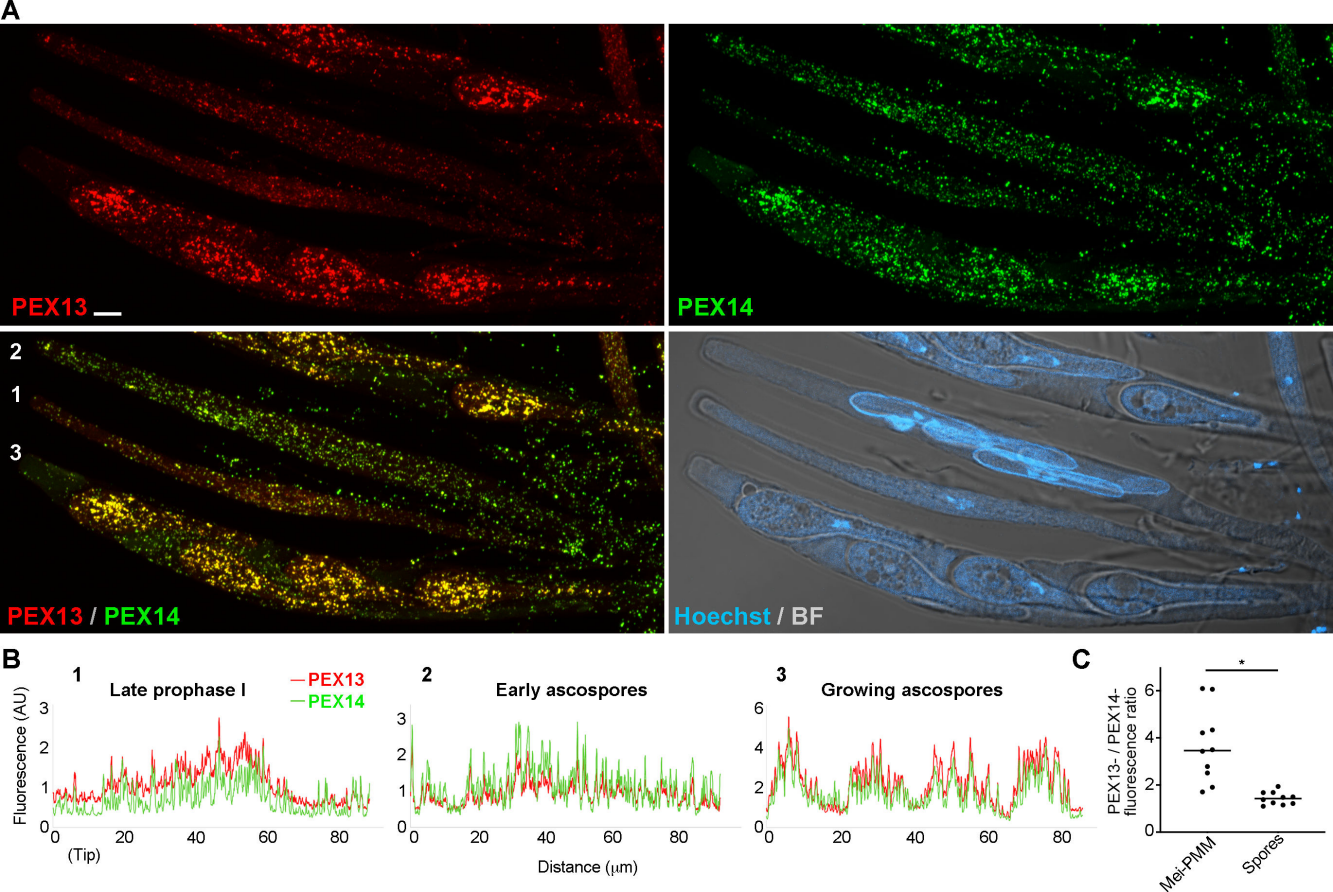
Localization of PEX13 and PEX14 during sexual development. (**A**) Compared localizations of PEX13 and PEX14 in asci at different developmental stages. Numbers indicate progressive developmental stages: (1) late prophase I (2), early ascospores, and (3) growing ascospores. Images show maximum-intensity projections of z-series through the entire cell volumes. DNA was stained by Hoechst. Scale bar, 5 μm. (**B**) Line scan graphs of the fluorescence intensity profile for PEX13-mCherry (red) and PEX14-GFP (green) channels along the asci indicated in (**A**). AU, Arbitrary units. (**C**) Ratio of PEX13-mCherry- to PEX14-GFP-fluorescence in grown asci (i.e., between late meiosis I and post-meiotic mitosis) and during ascospore formation (spores). *P < 0.05 by *t*-test, *n* = 10.

Next, we analyzed PEX13 localization relative to PEX14 during sexual development and found that PEX14-GFP localized to discrete punctae mostly colocalizing with PEX13-mCherry. Most PEX13-stained peroxisomes were decorated by PEX14 in dikaryotic cells (croziers, Fig. 9A) and in early meiotic prophase I asci (Fig. 9B). However, during ascus elongation—along prophase I progression—we observed an increase in the number of PEX13-labeled peroxisomes that contained low or no detectable PEX14 staining (Fig. 9C, arrows, compare to Fig. 9B) We found that the ratio of PEX13- to PEX14-stained peroxisomes increased along prophase I progression in correlation with ascus size, resulting in an approximately twofold increase in elongated asci, as compared to early asci (Fig. 9D). We also observed that the relative amount of PEX13 to PEX14 was higher at this stage, as compared to subsequent stages (Fig. 10).

**Fig 11:**
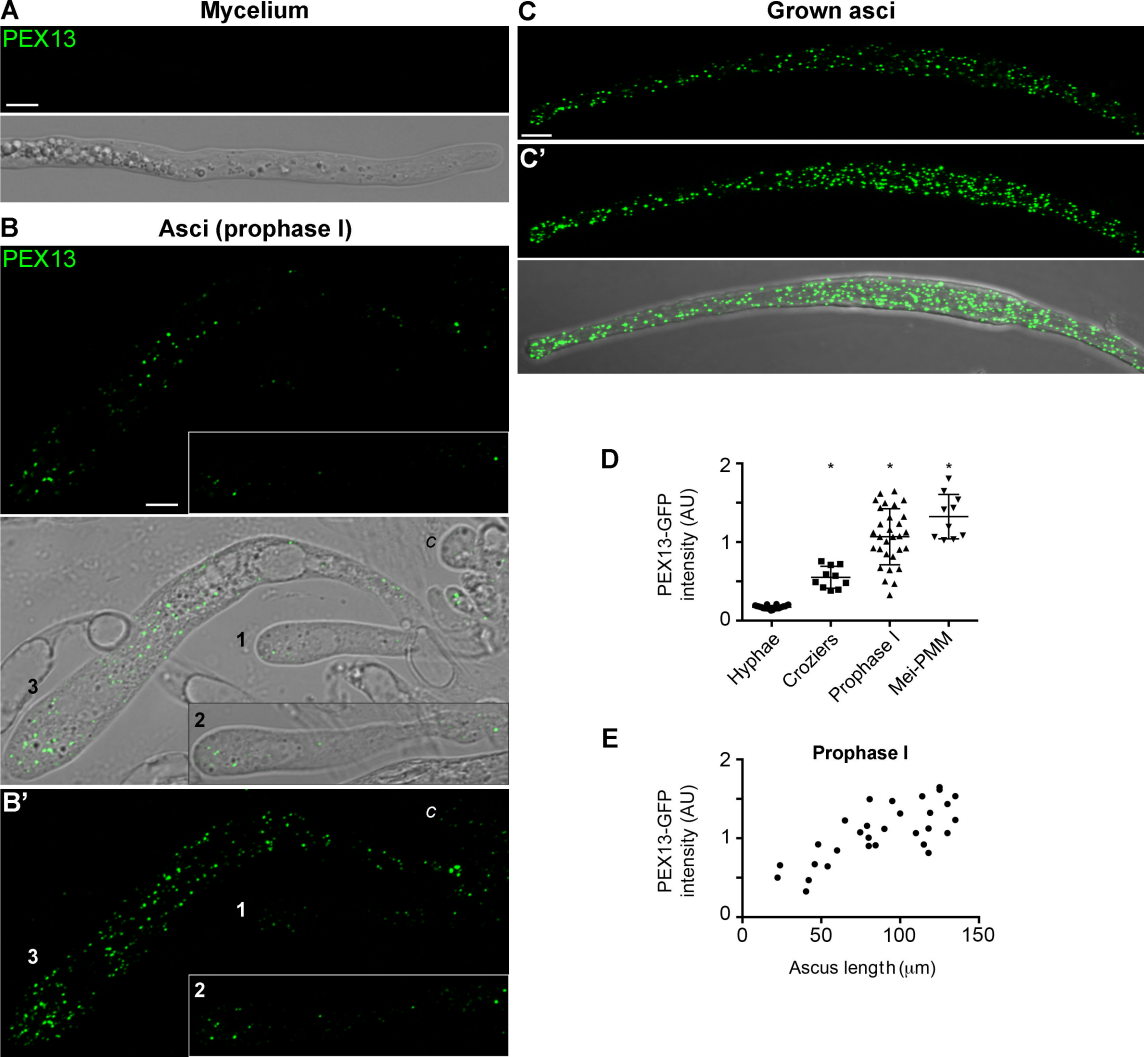
PEX13 levels increase during meiotic development. Localization of PEX13-GFP in mycelial hyphae (**A**) and at different stages of meiotic development (**B and C**). (**B**) Shows different stages of growing asci (along meiotic prophase I, three progressive stages are indicated by numbers, (*c*) indicates a crozier). (**C**) Shows an ascus after reaching its final length. (**A, B, C**) show single-plane images, (**B′ and C′**) show maximum-intensity projections of z-series of the entire volumes of the corresponding cells. Imaging parameters are equivalent to Fig. 3A and 7A. Scale bar, 5 μm. (**D**) Quantification of PEX13-GFP average fluorescence intensity in mycelial hyphae (*Hyphae*), dikaryotic croziers (*Croziers*), elongating asci (*Prophase I*, asci ranging between ≈10 and 150 μm in length), and grown asci (*Mei-PMM*, asci ≥150 μm in length, between late meiosis I and post-meiotic mitosis). Scatterplots show mean ± SD; *n* ≥ 10 from at least three independent experiments (i.e., 24 for hyphae, 10 for croziers and grown asci, 30 for prophase I asci). *P ≤ 0.001 relative to *hyphae* by one-way ANOVA with Tukey’s post hoc test. (**E**) Quantification of PEX13-GFP average fluorescence intensity in prophase I asci in relation to the ascus length. AU, Arbitrary units.

We noticed that the levels of PEX13-associated fluorescence were overall higher in sexual cycle cells than in mycelial hyphae, as well as in paraphyses—sterile vegetative hyphae interspersed between asci within the perithecium (Fig. 8C, arrows, compare to the FOX2 labeling). We then quantified PEX13-GFP fluorescence at different developmental stages. First, we analyzed PEX13 localization and fluorescence levels during meiotic development, as for mycelia (Fig. 11A through C). For this analysis, we initially categorized cells according to three developmental stages: (i) croziers (ii), elongating asci (ranging between ≈10 and 150 μm in length, corresponding to prophase I) (Fig. 11B), and (iii) grown asci (≥ 150 μm in length, corresponding to asci between late meiosis I and post-meiotic mitosis) (Fig. 11C). We found that PEX13-GFP fluorescence levels at these three stages were significantly higher than at mycelia (Fig. 11B and C, compared to Fig. 11A, quantitation in Fig. 11D) and that these levels were further incrementally increased along the progression of these stages (Fig. 11B through D). Moreover, we also found that PEX13-GFP levels increased along the progression of prophase I in correlation with ascus elongation (Fig. 11B and E).

**Fig 12:**
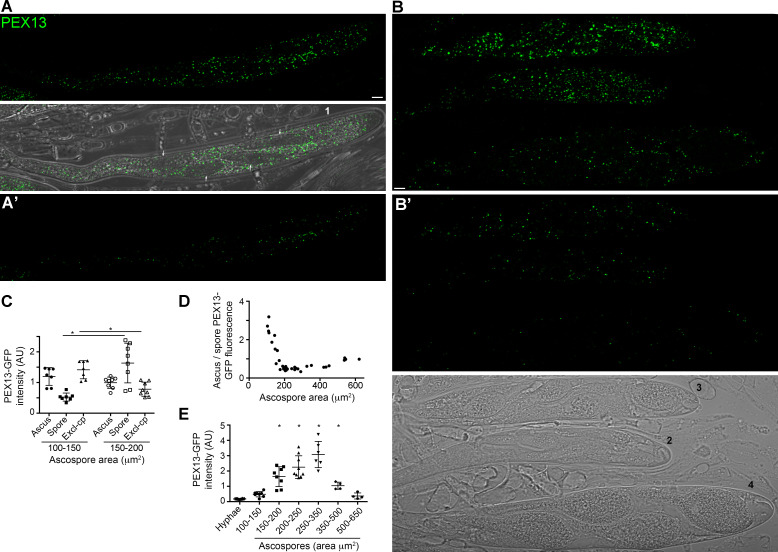
PEX13 levels change during ascospore differentiation. Localization of PEX13-GFP at distinct ascospore differentiation stages. Numbering indicates progressive stages of ascospore differentiation: (A, 1) shows early delineated ascospores, (B, 2–4) show progressive stages of ascospore growth. (**A**) and (**B**) show maximum-intensity projections of z-series through entire cells, (**A′ and B′**) show corresponding single-plane images. Arrows point to ascospores. Imaging parameters are equivalent to those of Fig. 3A, 7A, and 11. Scale bar, 5 μm. (**C**) Quantification of PEX13-GFP average fluorescence intensity in different ascus compartments during ascospore formation: *Ascus*, corresponds to the intensity of the entire ascus; *Spore*, to the average intensity of the four ascospores of an ascus, and *Excl-cp*, to the cytoplasm that is excluded from ascospores. Scatterplots show mean ± SD, *n* = 8. *P < 0.05 by one-way ANOVA with Tukey’s post hoc test. (**D**) Ratio of ascus to ascospore PEX13 fluorescence in relation to the ascospore size. (**E**) Quantification of PEX13-GFP average fluorescence intensity in mycelial hyphae (*Hyphae*) and ascospores at progressive differentiation stages, categorized according to their size. Scatterplots show mean ± SD; *n* = 24 for hyphae, eight for asci in the 100–350 μm^2^ categories, and 4 for the 350–650 μm^2^ categories (note that for each ascus the average fluorescence of its four ascospores was determined). *P < 0.05 relative to *Hyphae* by one-way ANOVA with Tukey’s post hoc test. AU, Arbitrary units.

Then, we analyzed PEX13 localization and abundance during ascospore differentiation (Fig. 12). Ascospore formation commences with the formation of new membranes inside the ascus, which encircle the nuclear products of meiosis together with a fraction of cytoplasmic contents. Upon closure of these membranes, the cytoplasm of the newly formed ascospores is separated from that of the original mother cell, which is later degraded by proteases released after permeabilization of the mother cell vacuole (49, 50). We found that at the very early stages of ascospore formation, PEX13-GFP fluorescence levels in the ascospore cytoplasm were highly reduced and were substantially lower than in the ascus cytoplasm that is excluded from ascospores (Fig. 12A, quantitation in C. See also Fig. 8D′). This suggests a removal or a high turnover of PEX13 in the peroxisomes that partition into ascospores. Afterward, PEX13-GFP levels inside ascospores increased, consistent with *de novo* production of PEX13 following ascospore formation while those of the cytoplasm remaining outside ascospores decreased (Fig. 12A through C). The ratio of ascus to ascospore PEX13 fluorescence rapidly decreased in the early steps of ascospore differentiation (Fig. 12D), showing a rapid increase in PEX13-GFP levels within ascospores relative to the total amount of PEX13-GFP.

In *P. anserina*, ascospores grow asymmetrically after being delimited and differentiate an expanding head and a slender tail portion. Ultimately, the tail is separated from the head by a septum and degenerates, while the head develops into the mature ascospore. Ascospore maturation also involves the gradual melanization of its cell wall (51). We found that PEX13-GFP fluorescence levels along this process increased in correlation to ascospore size, to then decrease to levels similar to those of ascospore initials, before the pigmentation and tail separation stages (Fig. 12B and E). These observations are consistent with differential modulation of PEX13 levels along ascospore differentiation.

## DISCUSSION

Here we analyzed the cell localization of the peroxin PEX13—a central component of the translocation channel through which peroxisomal proteins are imported into the organelle—along with *P. anserina* development. We found that PEX13 is maintained at low levels in vegetative cells by the activity of the peroxisome ubiquitination complex, in conjunction with the docking complex and the import receptor PEX5. We also discovered that PEX13 is differently regulated in the somatic and sexual phases of the life cycle and that it is also differentially regulated throughout meiotic development and at key sexual differentiation processes.

We discovered that the peroxisome ubiquitin ligase complex restrains the levels of PEX13 in *P. anserina* hyphae. These results are consistent with previous findings showing that *H. polymorpha* Pex13 is ubiquitinated in a Pex2-dependent manner and actively degraded likely *via* the proteasome (34). Likewise, Pex13 levels increase upon Pex2 depletion in human cells, where its removal involves the proteasome activity (52), and in *S. cerevisiae* Pex13 accumulates in ubiquitin ligase complex mutants defective in polyubiquitination (15). In addition, we found that PEX13 levels also increase in the absence of PEX4, similar to *H. polymorpha*. However, in *H. polymorpha,* the extent of Pex13 increase in *pex4* cells was much lower than in cells defective for the ubiquitin ligase complex (34)*,* whereas in *P. anserina* it was alike. Altogether, these observations are consistent with a model where *P. anserina* PEX13 is actively removed from vegetative cells, depending on the activity of the peroxisome ubiquitination complex. The involvement of PEX4 in this process could indicate that a cysteine-specific monoubiquitination is implicated in regulating PEX13 removal, which could contradict a proteasome degradation process involved in the regulation of PEX13 (53). However, Pex4 has also been found to be required for the polyubiquitination of peroxisome proteins, such as Pex20 in *Pichia pastoris* (38). Similar to *H. polymorpha* (34), we found that PEX14, PEX8, and PEX5 were also required to maintain low levels of PEX13, although apparently with a higher impact for PEX5 and PEX14 in *P. anserina*. In budding yeast, Pex13 interacts with both Pex14 and Pex8, while Pex8 bridges the docking peroxins to the RING-finger complex (39, 54). Therefore, in line with a ubiquitination-dependent regulation of PEX13 protein levels, delivery of PEX13 to the ubiquitination complex could depend on these interactions. The participation of PEX5 in this process is intriguing. The characterization of the peroxisome import machinery protein complexes shows that Pex13 is “loosely” associated with the docking complex (39, 55). Pex5 has been shown to contribute to the association of Pex13 with Pex14 (56). Moreover, recent evidence indicate that Pex5 could have a role in Pex14 insertion into the peroxisome membrane (57). Therefore, PEX5 could participate (directly and/or indirectly) in handing over Pex13 to the ubiquitination peroxins. Interestingly, Pex14 and Pex13 form a complex with cargo-loaded Pex5, which dissociates in the presence of cargo-unloaded Pex5 (58), it is thus tempting to speculate that Pex5 could signal for Pex13 removal after releasing its cargo in the peroxisome matrix.

We also found that the levels of PEX13 increase in different genetic backgrounds where peroxisome membranes are missing and that PEX13 associates with mitochondria in this context, indicating that PEX13 peroxisome membrane targeting is mandatory for its ubiquitination complex-dependent removal/regulation. How PEX13 associates with mitochondria was not addressed in this research. Nevertheless, our results are consistent with previous findings showing that in *Saccharomyces cerevisiae* several peroxisome proteins accumulate on mitochondria when peroxisomes are defective, most notably Pex13 (59). Pex13 localized to mitochondria in the absence of Pex19 or Pex3, and its mitochondrial levels were increased when the AAA ATPase Msp1—a mitochondrial extractase involved in removing mistargeted proteins—was simultaneously eliminated. In this context, Pex13 was integrated into the mitochondrial membrane. Moreover, PEX13 also displayed profuse mitochondria localization in PEX3-deficient human fibroblasts derived from Zellweger syndrome patients (59). On the other hand, it has been postulated that peroxisomes can be produced from vesicles that emanate from mitochondria and fuse with endoplasmic reticulum-derived vesicles, to produce peroxisome precursors (60). It is tempting to wonder whether PEX13 accumulating in mitochondria in peroxisome membrane-deficient cells represents the route through which PEX13 normally traffics to peroxisomes during peroxisome biogenesis. In line with this possibility, it is interesting that PEX13 was enriched at foci along mitochondria, similar to the peroxins that emanate from mitochondria into pre-peroxisomal precursors (60). Altogether, our findings show that PEX13 levels are finely regulated in vegetative hyphae depending on the activity of multiple components of the peroxisome matrix protein import machinery. Together with our previous research, our findings also show that different systems are involved in the removal, and possibly in the targeting, of the docking peroxins PEX13 and PEX14.

Despite its low levels, PEX13 is required for peroxisome matrix protein import in *P. anserina* hyphae (29). Pex13 possesses a tyrosine-/glycine-rich unstructured N-terminal domain that assembles into hydrogels (13) or droplet-like condensates (14), into which cargo-loaded Pex5 selectively partitions. Such condensates could define a nuclear pore-like phase at the peroxisome membrane through which protein import occurs. In yeasts, Pex13 undergoes dynamic changes at the peroxisome membrane, which involve the formation of transient foci correlating with cargo import and that could represent the formation of transient condensates acting as protein transport channels (14). The low PEX13 steady-state levels of *P. anserina* hyphae could reflect the transient and/or dynamic nature of the peroxisome protein translocation system in these cells. The increase in PEX13 abundance in sexual cells could indicate that the dynamics or the conformation of the translocation machinery is differently regulated during sexual development. Meiotic development in *P. anserina* relies on a specific configuration of the peroxisome protein import system, where Pex20 plays a central role (29, 32). The regulation of PEX13 abundance could be required to accommodate different (concentrations of) import receptors at defined developmental stages. We observed that PEX13 levels specifically increased along two key sexual differentiation processes, the differentiation of asci (meiocytes) and ascospores (gamete equivalents). Interestingly, we previously discovered that PEX14 is partially dispensable for peroxisome matrix protein import at these very stages (29). Moreover, during meiocyte differentiation, we now also observed a proliferation of PEX13-defined peroxisomes that lacked, or contained very low levels of, PEX14. Furthermore, our findings that PEX13 is required for meiocyte formation, but PEX14 is not, indicate a dikaryotic stage-specific configuration of the translocation machinery where PEX14 is dispensable, which is required to induce meiotic development. Altogether, these observations show that the peroxisome protein translocation machinery is finely regulated during meiotic development and is consistent with a foremost role for PEX13 in this process.

The regulation of PEX13 levels during development could also be related to the modulation of additional processes controlling peroxisome dynamics, like pexophagy. Recently, it has been shown that PEX13 prevents pexophagy in animal cells, and that pexophagy activation by different stimuli involves the proteasome-driven degradation of PEX13. It has therefore been proposed that PEX13 could participate in a peroxisome quality sensing system to regulate peroxisome homeostasis and removal (52). We have described that peroxisomes are eliminated from mature ascospores (46) at a subsequent stage to the phase where PEX13 levels decrease during ascospore differentiation. This peroxisome clearance could be mediated by pexophagy and constitute a quality control system involved in ascospore rejuvenation (61). Therefore, PEX13 could be involved in regulating pexophagy during ascospore differentiation, promoting the inheritance into ascospores of functional peroxisomes, and thereby contributing to meiotic rejuvenation.

## MATERIALS AND METHODS

### Strains and culture conditions

*P. anserina* strains used in this research are derived from the *S* wild-type background, and all analyzed strains were homokaryotic. All *PEX* single deletion mutants were previously generated (29, 31 – 33), and the strains expressing the distinct peroxisome fluorescent proteins of this research in the different genetic backgrounds were generated by genetic crosses. *P. anserina* was grown on an M2 minimal medium containing 1.1% dextrin as the sole carbon source. When required, dextrin was replaced by 0.05% oleic acid (plus 0.2% TWEEN 40 used as an emulsifier). M2 medium containing 0.55% dextrin and 2% agarose (instead of agar) was used for live cell imaging (MM medium). G (supplemented with 0.5% yeast extract) and RG media were used for ascospore germination and protoplast regeneration, respectively. Media were supplemented with phleomycin (25 μg mL^−1^), geneticin (G418 sulfate, 100 μg mL^−1^), nourseothricin (40 μg mL^−1^), or hygromycin B (70 μg mL^−1^) when required. Media composition can be consulted at http://podospora.i2bc.paris-saclay.fr.

### Plasmids, nucleic acid isolation, and transformation

*P. anserina* DNA isolation and transformation were performed as described in reference (62). The GFP-Hyg^R^ and mCherry-Hyg^R^ cassettes used for PEX13 tagging were obtained from plasmids pUC-GFP and pUC-mCherry, respectively (32).

### Tagging of PEX13

PEX13 was tagged by fusing in-frame the coding sequence of EGFP or mCherry to the 3′ end of *PEX13* open reading frame (ORF) at its endogenous locus. *PEX13* tagging constructs were generated by fusion PCR and consisted of the GFP-Hyg^R^ or the mCherry-Hyg^R^ cassette from pUC-GFP or pUC-mCherry, respectively (amplified with primers *pex13-lkt* and *pex13-hph*), preceded by the last 840 bp (excluding the stop codon) of *PEX13* ORF (amplified with primers *pex13-orf-F* and *lkt-pex13*) and followed by 819 bp of DNA downstream *PEX13* stop codon (amplified with primers *pex13-3F* and *pex13-3R*). The resulting constructs were cloned into pGEM-T Easy Vector (Promega, Madison, WI, USA) to yield plasmids pFS05 (*PEX13::GFP*) and pBA01 (*PEX13::mCherry*), respectively. *PEX13* tagging constructs were then PCR-amplified (with primers *pex13-orf-F* and *pex13-3R*), gel-purified, and used to transform protoplasts of a *Δku70* strain (63). Randomly selected Hygromycin-resistant (Hyg^R^) transformants were crossed to the wild type, and the Hyg^R^ marker was recovered in the *KU70^+^* genetic background in a monokaryotic context. All plasmids were verified by sequencing. *PEX13* tagging was verified by PCR analyses and by sequencing. Sequences of the oligonucleotide primers used are as follows: *pex13-lkt* (GAAGTCAACGTTCCAATCTGGTGACGGTGCTGGTTTA), *pex13-hph* (GTATGACA TTGGCCTCCTTTATTCCTTTGCCCTCGGA), *pex13-orf-F* (TTACATGGCCACGCATT CCAGCTTC), *lkt-pex13* (TAAACCAGCACCGTCACCAGATTGGAACGTTGACTTC), *pex13-3F* (TCCGAGGGCAAAGGAATAAAGGAGGCCAATGTCATAC), and *pex13-3R* (ACACACCCTTCC TCGTCACCACAC).

### Cytological analyses

Live cell imaging was done on whole individual *P. anserina* colonies grown for 24 h on MM as described previously (32). Sexual reproduction cells were fixed with paraformaldehyde (7.4%) and processed for imaging as previously described (64). Peroxisomes were visualized following fluorescent-labeled versions of FOX2 (mCherry or GFP) or PEX14 (GFP), expressed from their respective endogenous loci (32, 48). Mitochondria were stained by MitoTracker Red CMXRos (0.5 µM) (Molecular Probes, Eugene, OR), and nuclei were stained using either DAPI (4′,6-diamidino-2-phenylindole) (0.5 µg mL^−1^) (Molecular Probes) or Hoechst 33342 (2′-[4-ethoxyphenyl]−5-[4-methyl-1-piperazinyl]−2,5′-bi-1H-benzimidazole trihydrochloride trihydrate) (6.6 µg mL^−1^) (Molecular Probes). The colocalization analyses and the quantification of PEX13-GFP fluorescence levels in mycelia were done on confocal micrographs of the mid-plane of the apical segment of growing leading hyphae. For each hypha, the periphery of the hyphal region extending 80 μm from the tip was outlined and defined as a Region Of Interest (ROI). For the colocalization analyses, ROI thresholds were set with the Costes method, and Pearson’s coefficients were determined with the corresponding algorithm in ZEN 2012. For each strain, 21 hyphae issued from three biological replicates were analyzed. For PEX13-GFP fluorescence quantification, the intensity statistics of each ROI were calculated on ImageJ. The average fluorescence intensity was defined as the mean gray value of the corresponding ROI, and the background intensity was subtracted by tracing an equivalent ROI in an adjacent area with no sample signal. For each strain, 24 hyphae issued from three biological replicates (eight hyphae/replicate) were analyzed. Statistical significance was determined by two-way ANOVA with Tukey’s multiple comparisons test. Quantification of PEX13-GFP fluorescence during sexual development was done by an equivalent procedure. Sexual cycle cells were obtained from perithecia issued from *PEX13::GFP* homozygous crosses 3 days after fertilization. Sexual cells were dissected under a stereoscope, recovered in TPS buffer (5 mM Na_2_HPO_4_, 45 mM KH_2_PO_4_, 0.6 M sucrose), and immediately observed in a controlled temperature environment (see below). ROIs were defined as the area of the entire sexual cells. For asci containing ascospores, we determined the average fluorescence intensity of each ascospore of the ascus and the entire ascus. For early ascospore differentiation stages, we also determined the average fluorescence intensity of the ascus cytoplasm that is excluded from ascospores. Overall, 10 croziers, 40 meiotic asci, and 40 ascospore-containing asci issued from five independent experiments were analyzed. Statistical significance was determined by one-way ANOVA with Tukey’s multiple comparisons test. To quantify the number of peroxisomes containing PEX13 and PEX14, the area of *PEX13::mCherry PEX14::GFP* asci was defined as an ROI; for each channel (GFP, mCherry), the ROI threshold was set using the IsoData algorithm in ImageJ, and the peroxisome number was calculated with the *Analyze particles* function. 26 asci issued from five independent biological replicates were analyzed.

### Microscopy

Microscopy was performed on a Zeiss LSM800 inverted laser scanning confocal microscope using a Plan-Apochromat 63×/1.4 oil immersion objective, and 405-, 488- and 561-nm laser lines. Bright-field images were obtained with an Electronically Switchable Illumination and Detection (ESID) module. Live-cell imaging was performed in a similar system equipped with a controlled temperature chamber at 27C. For three-dimensional (3D) imaging, z-section images were acquired at 0.3- to 0.4-μm intervals through entire cell volumes. For PEX13-GFP detection, three imaging parameters were used: (i) laser power 0.3%, detector gain 662V (Fig. 3A, 7A, 11 and 12), (ii) laser 1.0%, detector gain 662V (Fig. 5), and (iii) laser 2.0%, detector gain 755V (Fig. 2A and 7B). For multi-channel imaging, images from all channels were collected simultaneously. Images were processed using ZEN 2012 (Carl Zeiss, Jena, Germany) or ImageJ (FIJI) (NIH, Bethesda, Maryland) software.

### Western blot analyses

Mycelial colonies were grown for 24 h at 27C on M2 plates topped with cellophane. For each plate (~30 colonies), the harvested mycelium was recovered on 500 µL of extraction buffer (25 mM Tris-HCl pH7.5, 150 mM NaCl, 0.5% NP40, 1 mM PMSF, 1 mM DTT, 1 × SigmaFast Protease Inhibitor) and added with 167 µL of 50% trichloroacetic acid. After 60 min at −75C, the samples were centrifugated (16,900 × *g,* 15 min), and the pellet was washed twice with 1 mL of cold 80% acetone by centrifugation (16,900 × *g*, 5 min), and air-dried. The proteins were recovered from the pellet with 100 µL of 0.1 NaOH, 1% sodium dodecyl sulfate (SDS), and 20µL of 6 × SDS loading buffer. Samples were boiled for 5 min, and 8 µL of each sample was loaded per well for an SDS-polyacrylamide gel electrophoresis. For Western blot analyses, GFP was detected with a monoclonal mouse anti-GFP antibody (B2, Santa Cruz Biotechnology, SC-9996) and GAPDH (glyceraldehyde 3-phosphate dehydrogenase) with a rabbit anti-GAPDH antibody (GeneTex, GTX100118), using HRP-conjugated anti-mouse (Jackson ImmunoResearch, 115–035-003) or anti-rabbit (Invitrogen, 31460) secondary antibodies, and the SuperSignal West Pico PLUS Chemiluminescent Substrate (Thermo Scientific). Images were captured with a C-DiGit Chemiluminescence Scanner (LI-COR), and the intensity of the protein bands was quantified on ImageJ.
